# Targeting the *Mycobacterium tuberculosis* Stringent Response as a Strategy for Shortening Tuberculosis Treatment

**DOI:** 10.3389/fmicb.2021.744167

**Published:** 2021-10-07

**Authors:** Carina Danchik, Siqing Wang, Petros C. Karakousis

**Affiliations:** Division of Infectious Diseases, Department of Medicine, Johns Hopkins School of Medicine, Baltimore, MD, United States

**Keywords:** *Mycobacterium tuberculosis*, stringent response, antibiotic tolerance, (p)ppGpp, hyperphosphorylated guanosine, inorganic polyphosphate, small molecule inhibitor, vaccination

## Abstract

The stringent response is well conserved across bacterial species and is a key pathway involved both in bacterial survival and virulence and in the induction of antibiotic tolerance in Mycobacteria. It is mediated by the alarmone (p)ppGpp and the regulatory molecule inorganic polyphosphate in response to stress conditions such as nutrient starvation. Efforts to pharmacologically target various components of the stringent response have shown promise in modulating mycobacterial virulence and antibiotic tolerance. In this review, we summarize the current understanding of the stringent response and its role in virulence and tolerance in Mycobacteria, including evidence that targeting this pathway could have therapeutic benefit.

## Introduction: the Need for New TB Treatment-Shortening Strategies

Current treatments for tuberculosis (TB) are lengthy and burdensome. Medical non-adherence and the continuing emergence of multi drug-resistant *Mycobacterium tuberculosis* strains contribute to the frequent failure of current antibiotic therapies to clear *M. tuberculosis* infection. The long duration required for curative TB treatment reflects the presence of a small population of bacteria characterized by antibiotic tolerance. Multiple strategies have been proposed to enhance the antibacterial activity of currently available antibiotics, thereby shortening the time required to achieve a stable cure, including boosting host defense pathways or inhibiting factors required for *M. tuberculosis* persistence and antibiotic tolerance, such as the stringent response (Kana et al., 2014; Frank et al., 2019).

### Antibiotic Resistance, Persistence, and Tolerance

Bacteria have evolved numerous strategies to evade killing by antibiotics. Reduced antibiotic susceptibility may result from genetic or phenotypic alterations and can be classified as resistance, persistence, or tolerance. Although each is associated with reduced antibiotic activity, it is important to distinguish among these three separate phenomena. A clear understanding of the diverse molecular pathways through which bacteria can become unresponsive to antibiotics is critical for the development of novel and more effective therapeutic approaches.

Antibiotic resistance is the most well-understood mechanism of bacterial insensitivity. The term applies to an entire bacterial population which exhibits an increase in the minimum inhibitory concentration of a particular antibiotic under optimal bacterial growth conditions. Antibiotic resistance may arise from mutations in genes encoding antibiotic targets or antibiotic-activating enzymes, mutations leading to increased enzymatic degradation of or reduced cell permeability to the antibiotic, or from increased activity of antibiotic efflux pumps (Hoffman, 2001; Karakousis, 2009; Westblade et al., 2020).

Antibiotic tolerance and persistence are both used to describe reversible states of broad insusceptibility to antibiotics. Although their usage in the literature remains ambiguous at times, antibiotic tolerance generally is used to describe an entire population of bacteria, while persistence refers to a subpopulation of phenotypically distinct bacteria within a clonal population. Both phenomena are frequently associated with a slowly growing or dormant state (Balaban et al., 2013; Maisonneuve and Gerdes, 2014).

Persistence is a non-heritable state in which a small proportion of bacteria in a clonal population can survive transient antibiotic exposure. This is characterized by a biphasic killing curve in which most of the bacterial population is killed rapidly, leaving behind a small number of persister bacteria which are insensitive to antibiotics and are killed at a much slower rate (Maisonneuve and Gerdes, 2014; Brauner et al., 2016). Persister cells may arise in the absence of any specific stimuli, perhaps due to stochastic differences in expression of specific genes, or may result from exposure to stress conditions. Persister cells are genetically identical to the rest of the bacterial population, and the antibiotic susceptibility patterns of daughter cells mirror those of the original parental population. Persistence has been attributed to reduced growth rate, metabolism, and protein synthesis as well as to the activity of toxin–antitoxin systems (Dahl et al., 2003; Sala et al., 2014; Harms et al., 2016; Talwar et al., 2020).

A wide array of stresses can induce antibiotic tolerance, including hypoxia, nitric oxide, and nutrient starvation (Gibson et al., 2018). Antibiotic tolerance is mediated by a variety of mechanisms, including the stringent response, discussed in more detail below, and drug efflux pumps (Goossens et al., 2021). Antibiotic tolerance may have a phenotypic or genetic basis and is often associated with an altered transcriptomic and metabolic state (Dahl et al., 2003; Dutta et al., 2019; Goossens et al., 2021). Additionally, actively dividing *M. tuberculosis* is able to develop antibiotic tolerance within macrophages through the activity of its efflux pumps (Adams et al., 2011, 2014; Szumowski et al., 2013; Black et al., 2014; Jang et al., 2017). These pumps can export antibiotics, reducing their intracellular concentrations and antibacterial effects, and pharmacological inhibition of the efflux pumps prevents the development of this macrophage-induced tolerance (Adams et al., 2011, 2014; Jang et al., 2017).

Although antibiotic resistance, tolerance, and persistence may reflect distinct phenomena, they may also be interrelated. Previous work has shown that bacterial mutations accumulate more rapidly under persistence- and tolerance-inducing stress conditions (Balaban et al., 2013). Such mutations can confer resistance to antibiotics, thus promoting the survival of tolerant bacteria (Levin-Reisman et al., 2017).

### The Stringent Response

The stringent response is a conserved bacterial adaptation to nutrient starvation and other stress conditions. It is mediated by tetra- or penta-phosphorylated guanosine [(p)ppGpp] and inorganic polyphosphate [poly(P)]. Accumulation of these two regulatory molecules in *E. coli* leads to downregulation of growth-related pathways through altered transcription and metabolism (Traxler et al., 2008). In *M. tuberculosis*, the stringent response enables bacteria to survive host defenses by inducing metabolic quiescence, thereby contributing to long-term mycobacterial survival and virulence (Primm et al., 2000). By reducing mycobacterial growth and metabolism, the stringent response also contributes to antibiotic tolerance by suppressing the activity of numerous antibiotic targets.

#### Rel_Mtb_ and (p)ppGpp

The production and metabolism of (p)ppGpp are mediated by members of the RelA/SpoT homology (RSH)-type protein family, which are conserved across bacterial species. In *E. coli* and some other gram-negative bacteria, (p)ppGpp synthesis and hydrolysis are regulated by two separate enzymes, RelA and SpoT, respectively, where RelA is a (p)ppGpp synthase and SpoT is a bifunctional protein with both (p)ppGpp synthesis and hydrolysis activities (Ronneau and Hallez, 2019). *M. tuberculosis* contains a single bifunctional RSH enzyme, designated Rel_Mtb_, with dual (p)ppGpp synthesis and hydrolysis activities (Avarbock et al., 1999). The constitutive activity of Rel_Mtb_ maintains (p)ppGpp at a basal level and is required for bacterial growth and biofilm formation *in vitro* and *in vivo* (Weiss and Stallings, 2013).

The opposing activities of Rel_Mtb_ are contained in distinct regions of the protein. Rel_Mtb_ is a 738-amino acid enzyme, with catalytic domains in the N-terminal region and regulatory domains in the C-terminal region (Avarbock et al., 1999, 2005). In the N-terminal region (AA 1–394), residues 1–203 exhibit (p)ppGpp hydrolysis activity, and residues 87–394 exhibit (p)ppGpp synthesis activity, while the C-terminal regulatory domains are confined to residues 395–738 (Avarbock et al., 2005; Singal et al., 2017). The synthase domain catalyzes the transfer of the 5′-beta, gamma-pyrophosphate group from ATP to the 3’ OH group of GDP or GTP, producing ppGpp or pppGpp, respectively. The hydrolysis domain catalyzes the opposite reaction, i.e., hydrolysis of the pyrophosphate group on (p)ppGpp, producing GDP or GTP (Avarbock et al., 2005).

During amino acid deprivation, uncharged tRNA accumulates and competes with charged tRNA for the ribosome aminoacyl (A) site, giving rise to the Rel_Mtb_ activating complex, which is comprised of uncharged tRNA, ribosomes, and cognate mRNA (Avarbock et al., 2000). This complex stimulates Rel_Mtb_ synthase activity to produce (p)ppGpp. Conversely, under conditions of amino acid abundance, charged tRNA outcompetes uncharged tRNA at the ribosome A-site, suppressing (p)ppGpp synthesis (Avarbock et al., 2000).

(p)ppGpp is a global regulator of the stress and starvation response in bacteria. In *E. coli*, a basal level of (p)ppGpp is required to maintain normal low levels of the alternative sigma factor RpoS (Battesti et al., 2011). Under stress conditions, elevated (p)ppGpp levels induce production and reduce degradation of RpoS, which, in turn, regulates expression of 10% of the *E. coli* genome through direct or indirect interaction with RNA polymerase. This altered transcription yields a stationary phase-like phenotype marked by slowed growth and stress resistance (Battesti et al., 2011). In *M. tuberculosis*, a total of 159 genes, including many encoding virulence factors, important antigens, and proteins involved in transcription and translation, are regulated by (p)ppGpp expression (Dahl et al., 2003).

#### Inorganic Polyphosphate [poly(P)]

Inorganic polyphosphate [poly(P)], a linear polymer consisting of up to hundreds of phosphate residues, is ubiquitous across bacterial species and plays diverse roles in growth mediation, stress responses, and virulence (Rao et al., 2009). Poly(P) metabolism is controlled by polyphosphate kinases (PPKs) and exopolyphosphatases (PPXs), which are responsible for synthesis and hydrolysis, respectively. PPK1 (Rv2984), the primary polyphosphate kinase in *M. tuberculosis*, catalyzes the formation and elongation of a poly(P) chain using the gamma-phosphate from ATP. *Ppk1* transcription is upregulated by the two-component system SenX3-RegX3 under phosphate-limited conditions, leading to increased poly(P) synthesis (Rifat et al., 2009; Sanyal et al., 2013; Namugenyi et al., 2017). PPK2 (Rv3232c), although historically annotated as a kinase due to its first described activity, is primarily responsible for poly(P) hydrolysis and may be more accurately characterized as a PPX. PPX1 (Rv0496) hydrolyzes short-chain poly(P), while PPX2 (Rv1026) hydrolyzes long-chain poly(P) (Sureka et al., 2007, 2009; Thayil et al., 2011; Chuang et al., 2015). Poly(P) levels increase during the stationary phase of growth and under various stress conditions, including upon exposure to antibiotics, and promote bacterial survival and virulence (Singh et al., 2013; Chuang et al., 2015). In *E. coli*, poly(P) binds and activates the ATP-dependent protease Lon, stimulating protein degradation to provide an alternative source of molecular building blocks during amino acid deprivation (Kuroda, 2006; Singh et al., 2013).

**Figure 1:**
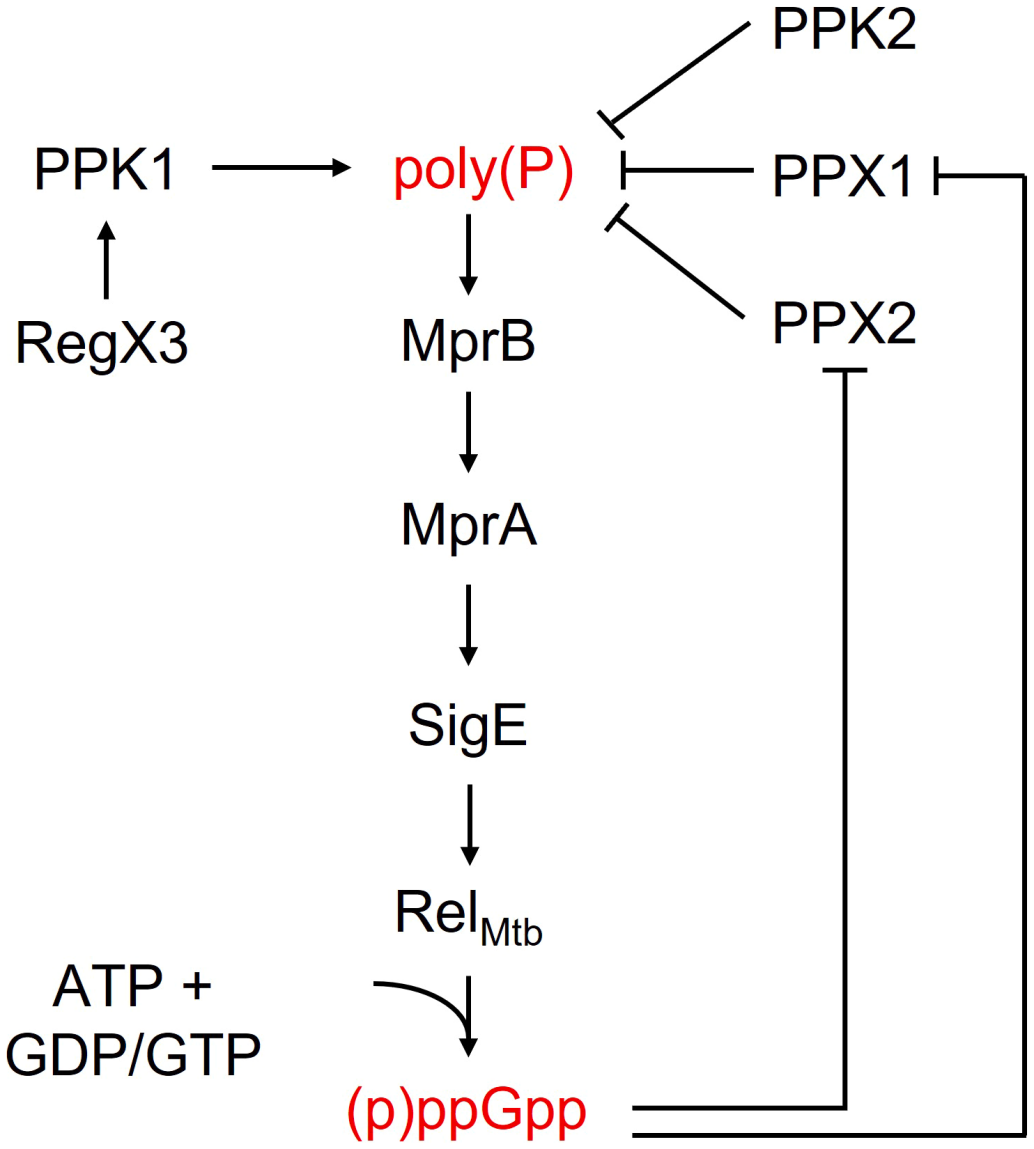
Activity and regulation of key bacterial stringent response factors.

The two stringent response molecules are also able to positively regulate each other (Figure 1). (p)ppGpp also binds to and inhibits PPX1 and PPX2, decreasing poly(P) degradation and leading to poly(P) accumulation (Choi et al., 2012; Chuang et al., 2015). Conversely, poly(P) induces (p)ppGpp synthesis through the *mprAB-sigE-rel_Mtb_* regulatory cascade (Sureka et al., 2007). In this cascade, poly(P) serves as the phosphate donor for autophosphorylation of the histidine kinase MprB and its subsequent phosphorylation of MprA. MprA then regulates the expression of its own *MprAB* operon as well as that of *sigE*, a sigma factor which promotes *rel_Mtb_* transcription (Sureka et al., 2007). Thus, both (p)ppGpp and poly(P) regulate each other through a positive feedback mechanism. The intracellular levels of these regulatory molecules are controlled primarily by the bifunctional RSH enzyme Rel_Mtb_ and by the various kinases and phosphatases involved in poly(P) metabolism and signaling pathways, which are critical for eliciting and maintaining the stringent response under stress conditions.

## The Role of the Stringent Response in *M. Tuberculosis* Virulence

The stringent response contributes to *M. tuberculosis* survival under physiologically relevant stress conditions and to virulence within the infected mammalian host. Disruption of Rel_Mtb_, PPK, or PPX leads to abnormal levels of (p)ppGpp, poly(P), and other cell metabolites, as well as to reduced viability and virulence (Primm et al., 2000; Dutta et al., 2019).

Deletion of *rel_Mtb_* is associated with defective *M. tuberculosis* survival under nutrient starvation and hypoxia conditions (Primm et al., 2000). Recently, a *rel_Mtb_*-deficient mutant was found to have an impaired ability to slow its replication rate or downregulate lipid metabolism during nutrient starvation, resulting in reduced viability (Dutta et al., 2019). Although aerosol infection of C57BL/6 mice with Δ*rel_Mtb_* resulted in normal initial bacterial growth and containment, the long-term survival of this strain in mouse lungs and spleens was severely impaired, and the histopathology of these organs was markedly reduced in mice infected with Δ*rel_Mtb_* relative to those infected with the isogenic wild-type strain (Dahl et al., 2003). Similarly, a *rel_Mtb_*-deficient mutant was found to have reduced extracellular survival in a murine hypoxic granuloma model of latent TB infection (Karakousis et al., 2004; Klinkenberg et al., 2008). Deletion of *rel_Mtb_* resulted in impaired initial *M. tuberculosis* growth and survival, as well as a striking absence of gross tubercle lesions and histological evidence of caseous granulomas in the lungs of infected guinea pigs (Klinkenberg et al., 2010). Finally, C3HeB/FeJ mice, which develop necrotic TB lung granulomas characterized by tissue hypoxia, had significantly prolonged survival after aerosol infection with ∆*rel_Mtb_* than after infection with the isogenic wild-type strain (Harper et al., 2012; Dutta et al., 2019).

Poly(P) accumulates in response to stress conditions, and disruption of the enzymes involved in poly(P) synthesis and hydrolysis affects the bacteria’s ability to adapt to these conditions. *M. tuberculosis* mutants deficient in PPX1, PPX2, and PPK2 have impaired hydrolysis activity and exhibit constitutively higher levels of intracellular poly(P) relative to wild type (Thayil et al., 2011; Chuang et al., 2013, 2016). Conversely, a PPK1-deficient mutant showed impaired synthesis activity and exhibited lower levels of poly(P) (Prusa et al., 2018). Poly(P)-accumulating strains deficient in PPX1, PPX2, and PPK2 were found to have defective biofilm formation (Chuang et al., 2015, 2016).

Poly(P) accumulation in *M. tuberculosis* is also associated with increased lipid oxidation and citrate cycle activity, as well as altered expression of genes involved in glycerol-3-phosphate (G3P) homeostasis, contributing to reduced intracellular G3P content in poly(P)-accumulating strains (Chuang et al., 2016). G3P can be used as a scaffold for phospholipid biosynthesis, and overexpression of G3P dehydrogenase in *E. coli* leads to reduced intracellular G3P levels and increased formation of persister cells following exposure to antibiotics (Spoering et al., 2006; Yao and Rock, 2013). Poly(P) accumulation also appears to alter peptidoglycan synthesis in *M. tuberculosis* (Chuang et al., 2016). A *ppx2* knockdown strain displayed altered cell wall thickness and permeability, and a *ppk2*-deficient mutant showed reduced Nile red staining relative to control strains (Chuang et al., 2015, 2016). Furthermore, a PPK2-deficient mutant was found to be more sensitive to the toxic hydroxy-1,4-naphthoquinone, plumbagin, relative to isogenic wild-type and complemented strains (Chuang et al., 2016).

Poly(P) accumulation also affects *M. tuberculosis* virulence *in vivo*. A *ppk2*-deficient mutant showed reduced survival at day 7 in activated and naive J774 macrophages relative to the wild-type strain, and naive macrophages showed increased expression of interleukin 2 (IL-2), IL-9, IL-10, IL-12p70, and gamma interferon (IFN-γ) following infection with the mutant relative to those infected with the wild type (Chuang et al., 2013). Consistent with a requirement of *ppk2* for full *M. tuberculosis* virulence *in vivo*, a *ppk2*-deficient mutant exhibited a significantly lower lung bacillary burden during acute murine infection compared to the control groups (Chuang et al., 2013). Similarly, a *ppx1*-deficient mutant showed a significant survival defect in activated human macrophages and reduced survival in the lungs of guinea pigs (Thayil et al., 2011). Furthermore, *M. tuberculosis* deficiency of the phosphate-specific transport PhoU orthologs, PhoY1 and PhoY2, led to increased transcription of *ppk1* in a RegX3-dependent manner, accumulation of poly(P) during log phase growth, and reduced survival relative to wild type in the lungs of mice 12weeks after infection (Namugenyi et al., 2017).

As in the case of poly(P) accumulation, poly(P) deficiency is also associated with reduced *M. tuberculosis* stress adaptation and virulence. Thus, compared to the wild-type strain, a *ppk1*-deficient mutant displayed a survival defect in response to nitrosative stress *in vitro* and in THP-1 macrophages, as well as a lower mycobacterial load and fewer necrotic granulomas in the lungs of guinea pigs at 10weeks post-infection (R. Singh et al., 2013). Taken together, these data suggest that poly(P) levels are tightly regulated in *M. tuberculosis*, and disturbance of poly(P) homeostasis is associated with a reduced stress resistance and virulence.

## The Role of the Stringent Response in *M. Tuberculosis* Antibiotic Tolerance

Perturbation of the *M. tuberculosis* stringent response leads to altered antibiotic susceptibility, suggesting that, in addition to stress adaptation and virulence, this pathway also plays a role in antibiotic tolerance.

Although deletion of the *rel_Mtb_* gene did not alter *M. tuberculosis* susceptibility to multiple antibiotics during logarithmic growth in nutrient-rich broth, the MBC of isoniazid was reduced 512-fold against Δ*rel_Mtb_* relative to the wild-type and complemented strains during nutrient starvation (Primm et al., 2000; Dutta et al., 2019). Furthermore, 2weeks of oral therapy with human-equivalent doses of isoniazid significantly reduced the lung bacillary burden of mice chronically infected with Δ*rel_Mtb_* (2.03 log_10_ CFU reduction) relative to those infected with control strains (0.11 log_10_ CFU reduction; *p*<0.0001; Dutta et al., 2019). These findings suggest that the stringent response is required for *M. tuberculosis* tolerance to bactericidal antibiotics during stress exposure *in vitro* and in animal tissues.

Dysregulation of poly(P) homeostasis also alters the antibiotic susceptibility profile of *M. tuberculosis*. Thus, a poly(P)-deficient *ppk1* deletion mutant showed increased susceptibility to several antibiotics, including isoniazid and levofloxacin (Singh et al., 2013). Alternatively, a poly(P)-accumulating Δ*phoY1* Δ*phoY2* double mutant was found to have increased susceptibility to rifampin *in vitro* and in mouse lungs during the chronic phase of injection, and this phenotype was not associated with altered cell wall permeability (Namugenyi et al., 2017). Two other poly(P)-accumulating strains, namely a *ppx2* knockdown mutant (Chuang et al., 2015) and a *ppk2* transposon mutant (Chuang et al., 2013), displayed reduced susceptibility to isoniazid, although the latter strain showed increased susceptibility to meropenem (Chuang et al., 2016). Another poly(P)-accumulating strain deficient in PPX1 showed increased susceptibility to clofazimine (Chuang et al., 2016).

Taken together, these data suggest that proper regulation of the stringent response is critical for antibiotic tolerance in *M. tuberculosis*.

## Targeting the Stringent Response as a Treatment Strategy

Since the stringent response is critical for *M. tuberculosis* stress adaptation, virulence, and antibiotic tolerance, targeting this pathway represents an attractive approach for improving TB treatment outcomes. The two main strategies proposed so far are inhibition *via* small molecules (summarized in Table 1) and priming of the host immune response *via* DNA vaccination.

**Table 1:** Small molecule inhibitors of bacterial stringent response factors.

Reference	Compound name	Target	Species	Structure
Dutta et al., 2019	X9	Rel_Mtb_	*M. tuberculosis*	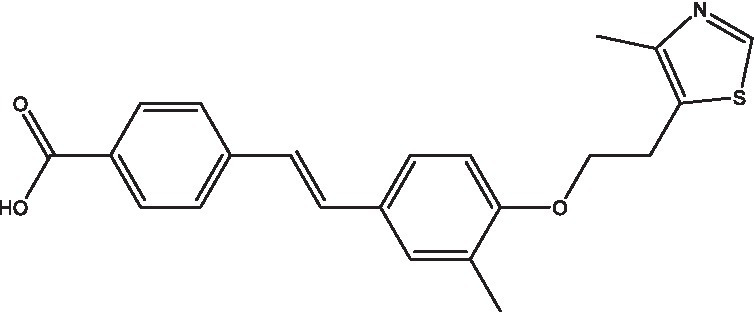
Njire et al., 2017	Pyrazinoic acid	Rv2783c	*M. smegmatis*, *M. tuberculosis*	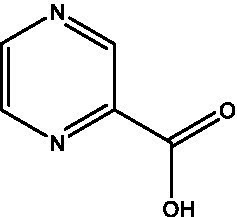
Singh et al., 2016	NSC-9037	PPK2	*M. tuberculosis*	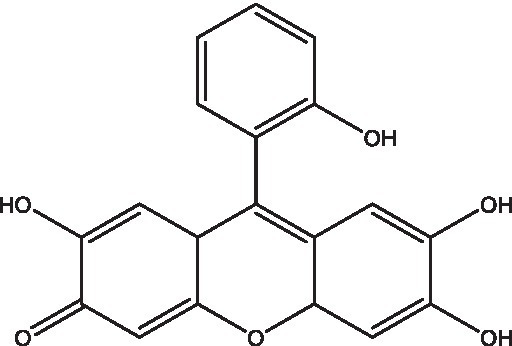
Singh et al., 2016	NSC-35676	PPK2	*M. tuberculosis*	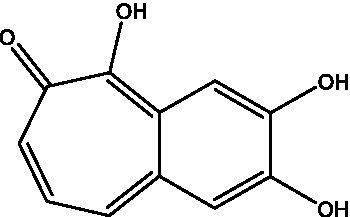
Syal et al., 2017a	Vitamin C	Rel_Mtb_	*M. smegmatis*	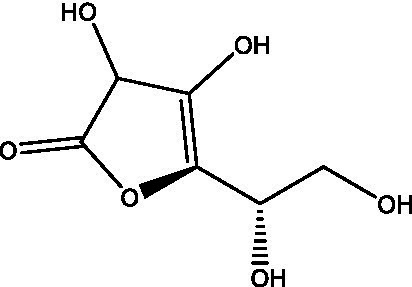
Syal et al., 2017b	Acetylated Relacin analog	Rel_Mtb_	*M. smegmatis*, *M. tuberculosis*	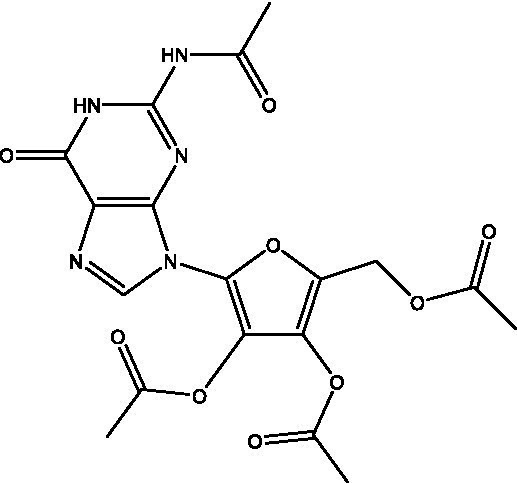
Syal et al., 2017b	Acetylated benzoylated Relacin analog	Rel_Mtb_	*M. smegmatis*, *M. tuberculosis*	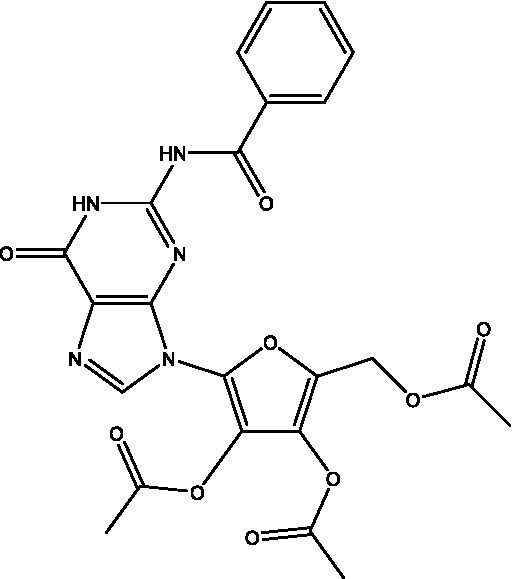
Tkachenko et al., 2021	DMNP	Rel_Mtb_	*M. smegmatis*	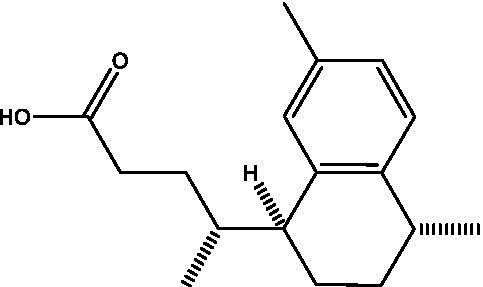
Wexselblatt et al., 2012	Relacin	Rel_Mtb_	*B. subtilis*, *D. radiodurans*	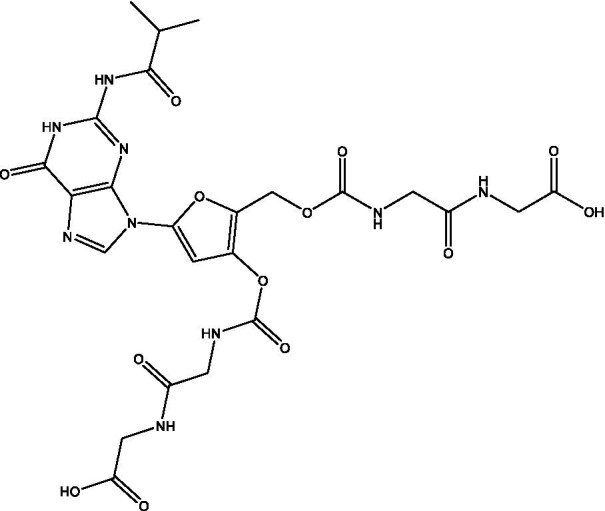
Wexselblatt et al., 2013	Relacin analog 2d	Rel_Mtb_	*B. subtilis*	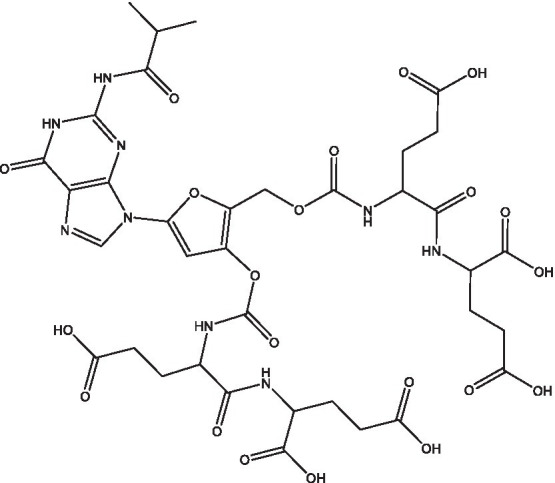

### Small Molecule Inhibitors of Stringent Response Factors

Disruption of the stringent response by perturbing poly(P) homeostasis has been proposed as a novel TB treatment strategy. To this end, inhibitors of PPK1 and PPK2 have been developed. A small *in silico* screen of 18 predicted inhibitors was performed for PPK1. The three lead compounds from this screen showed robust activity with inhibition constants ranging from 255 to 866nM (Shahbaaz et al., 2019). A high-throughput screen of 2,300 compounds for PPK2 inhibitors identified two lead compounds with >80% inhibition of enzyme activity at 100μM (M. Singh et al., 2016). A DNA aptamer with an IC_50_ of 40nM in a biochemical assay has also been designed for PPK2 (Shum et al., 2011). The finding that a *ppk2* transposon mutant displayed a 4-fold increase in the MIC of isoniazid relative to the wild-type and complemented strains is concerning, however, as this effect could counteract any potential benefits of incorporating a PPK2 inhibitor into current TB treatment regimens (Chuang et al., 2013; Singh et al., 2016).

Since regulation of (p)ppGpp levels is critical for the activation of the stringent response and for bacterial adaption to stress conditions, inhibition of the bifunctional Rel_Mtb_ protein has also been proposed as a treatment strategy (Wexselblatt et al., 2012; Dutta et al., 2019).

Multiple groups have sought to identify RelA inhibitors. The earliest of these inhibitors, Relacin, was identified using modeling based on the crystal structure of Rel/Spo from *Streptococcus equisimilis* (Wexselblatt et al., 2012). This small molecule was able to decrease (p)ppGpp levels and disrupt biofilm formation in *Bacillus subtilis*. It also inhibited sporulation in a dose-dependent manner (Wexselblatt et al., 2012). Deoxyguanosine-based analogs were subsequently designed to optimize the compound, and one of these had increased potency against RelA from *E. coli* (Wexselblatt et al., 2013). Two additional analogs (a benzoylated and an acetylated benzoylated version) were also synthesized and tested for their ability to decrease levels of (p)ppGpp in *M. smegmatis* (Syal et al., 2017b). The acetylated benzoylated analog had an IC_50_ of ~40μM and caused elongation of the cells (Syal et al., 2017b), which is in line with previous morphological characterization of mycobacteria lacking *rel* (Gupta et al., 2016). This compound also decreased long-term survival of *M. smegmatis* and inhibited biofilm formation in both *M. smegmatis* and *M. tuberculosis* (Syal et al., 2017b). The activity of Relacin and its analogs against multiple bacterial species suggest that these Rel inhibitors could have broad applications as antibiotics.

A high-throughput strategy using a truncated recombinant version of Rel_Mtb_ and a fluorescence polarization assay to screen a library of over 2million compounds for enzymatic inhibition of (p)ppGpp synthesis activity yielded 178 hits with favorable physicochemical properties (Dutta et al., 2019). In whole-cell assays, one of these hits (X9) dosed at 2μM induced significant killing of nutrient-starved, wild-type *M. tuberculosis*, phenocopying the survival defect of the untreated Δ*rel_Mtb_* mutant during nutrient starvation, and hypersensitized the nutrient-starved wild type to cell wall-active agents, decreasing the MBC of isoniazid 16-fold. These findings suggest that pharmacological inhibition of the *M. tuberculosis* stringent response results in direct mycobacterial killing under growth-limiting conditions, as well as reversal of tolerance to cell wall-active agents.

Several natural products have also demonstrated anti-Rel activity. Vitamin C inhibits the (p)ppGpp synthesis activity of Rel and decreases cell viability and biofilm formation in *M. smegmatis* (Syal et al., 2017a). The compound, 4-(4,7-dimethyl-1,2,3,4-tetrahydronaphthalene-1-yl)pentanoic acid (DMNP), a derivative of erogorgiaene, was identified as an inhibitor of both Rel_Msm_ and RelZ in *M. smegmatis* (Tkachenko et al., 2021). DMNP showed antibacterial activity against *M. smegmatis* in a whole cell assay, and overexpression of either Rel_Msm_ or RelZ reduced the compound’s antibiotic effect and increased the number of persisters. DMNP further inhibited the (p)ppGpp synthase activity of purified Rel_Msm_ and showed similar predicted binding sites for Rel_Msm_ or RelZ in molecular docking simulations (Tkachenko et al., 2021).

One FDA-approved antitubercular drug appears to target the *M. tuberculosis* stringent response, among other putative mechanisms of action. Pyrazinamide is a critical component of current treatment regimens for drug-susceptible TB and is formulated as a prodrug which is converted by *M. tuberculosis* pyrazinamidase to pyrazinoic acid. Pyrazinoic acid, but not the prodrug, was found to inhibit Rv2783c, which is involved in the RNA degradosome, a multi-enzyme complex important in RNA processing and mRNA degradation, with a K_d1_ of 1.05mM and a K_d2_ of 3.17mM (Njire et al., 2017). This binding was completely abolished in an D67N mutant and in enzyme isolated from naturally pyrazinamide-resistant *M. smegmatis*. In addition to its role in mRNA processing and degradation, Rv2783c has multifunctional activities, including polymerization and phosphorolysis of single-stranded DNA and (p)ppGpp hydrolysis *in vitro*. Each of these activities can be inhibited by pyrazinoic acid. The point mutation which eliminated pyrazinoic acid binding to Rv2783c resulted in a 5-fold increase in the MIC of pyrazinamide against *M. tuberculosis*, further underscoring the importance of Rv2783c as a target for this antitubercular drug (Njire et al., 2017).

### Therapeutic DNA Vaccination Targeting Stringent Response Factors

In addition to pharmacological modulation of the stringent response, recent work has focused on potentiating host immunity to *M. tuberculosis* stringent response factors as a treatment-shortening strategy since this pathway is important for long-term mycobacterial survival in host tissues, and is induced during chronic TB infection in the lungs of mice (Talaat et al., 2004).

Intramuscular immunization of mice with a DNA vaccine targeting four different *M. tuberculosis* stringent response genes (*rel_Mtb_*, *sigE*, *ppk2*, and *ppx*) induced significant antigen-specific IgG responses, antigen-specific IFN-γ-producing CD4+ T cells, and *rel_Mtb_*-specific TNF-α-producing CD4+ T cells (Chuang et al., 2016). Although DNA vaccine containing all four stringent response genes was not protective against aerosol challenge with virulent *M. tuberculosis*, it enhanced the activity of isoniazid in a mouse model of chronic infection, significantly reducing the *M. tuberculosis* burden in the lungs relative to mice receiving the control DNA vaccine (Chuang et al., 2016).

Of the four stringent response genes included in the above vaccine, isoniazid exposure of *M. tuberculosis*-infected macrophages increased expression *of rel_Mtb_* most robustly (Chuang et al., 2020), prompting generation of a DNA vaccine targeting this gene alone. The *rel_Mtb_* DNA vaccine conferred increased susceptibility of *M. tuberculosis* to isoniazid relative to the control DNA vaccine in a mouse model of chronic TB infection and reduced regrowth of bacteria after cessation of antibiotic treatment (Chuang et al., 2020). Thus, Rel_Mtb_ appears to be an important persistence antigen, which can be targeted by cellular immune responses in conjunction with antitubercular drugs to assist with clearance of *M. tuberculosis* infection from host tissues.

## Discussion

The stringent response is involved in bacterial adaptation to stress, contributing to survival, virulence, and drug tolerance within the host. (p)ppGpp and poly(P) play essential roles in mediating the stringent response, and mutations in *M. tuberculosis* proteins involved in their metabolism lead to reduced mycobacterial virulence and enhanced sensitivity to antibiotics.

Inhibition of stringent response factors such as Rel_Mtb_ shows promise as an anti-tuberculosis strategy. While there are numerous reports of small molecule inhibitors of stringent response factors, further medicinal chemistry campaigns are required to optimize the activity, bioavailability, and pharmacokinetics of these compounds. Additionally, relapse studies in clinically relevant animal models are needed to determine whether the addition of a stringent response inhibitor to current TB treatment regimens can shorten the time required to achieve a stable cure. Based on the established role of the stringent response in *M. tuberculosis* antibiotic tolerance, we predict that addition of a stringent response modulator, whether a small molecule inhibitor or a therapeutic vaccine enhancing antigen-specific T cell responses, may reduce the number of persisters, thereby promoting more rapid eradication of infection from host tissues.

Novel strategies targeting the stringent response pathway are predicted to have treatment-shortening potential against both drug-susceptible and drug-resistant *M. tuberculosis*. This is a particularly important consideration for multidrug-resistant (MDR) TB and extensively drug-resistant (XDR) TB, since standard regimens to treat these infections have markedly reduced efficacy and require more than 18months of continuous therapy. Thus, the stringent response represents a promising target for the development of novel antimycobacterial strategies.

## Author Contributions

CD and PK designed and directed the project. CD and SW performed the literature search. CD, SW, and PK wrote the article. All authors contributed to the article and approved the submitted version.

## Funding

This work was supported by NIH grants R01AI148710, R21AI140860, and K24AI143447 to PK. The funding source had no role in the design and conduct of the study.

## Conflict of Interest

The authors declare that the research was conducted in the absence of any commercial or financial relationships that could be construed as a potential conflict of interest.

## Publisher’s Note

All claims expressed in this article are solely those of the authors and do not necessarily represent those of their affiliated organizations, or those of the publisher, the editors and the reviewers. Any product that may be evaluated in this article, or claim that may be made by its manufacturer, is not guaranteed or endorsed by the publisher.
